# The Intimate Relationship between Vestibular Migraine and Meniere Disease: A Review of Pathogenesis and Presentation

**DOI:** 10.1155/2016/3182735

**Published:** 2016-08-29

**Authors:** Yuan F. Liu, Helen Xu

**Affiliations:** Department of Otolaryngology, Head and Neck Surgery, Loma Linda University Medical Center, Loma Linda, CA, USA

## Abstract

Vestibular migraine (VM) has only recently been recognized as a distinct disease entity. One reason is that its symptoms overlap greatly with those of other vestibular disorders, especially Meniere disease (MD). The pathophysiology of neither VM nor MD is entirely elucidated. However, there are many theories linking migraine to both disorders. We reviewed the current understanding of migraine, VM, and MD and described how VM and MD are similar or different from each other in terms of pathophysiology and presentation, including hypotheses that the two share a common etiology and/or are variants of the same disease.

## 1. Introduction

Vestibular migraine (VM) as a distinct disease entity came into the hesitant eye of the medical community no earlier than the advent of the new millennia [1]. Although monikers fitting of the disorder, migrainous vertigo, migraine-associated dizziness, benign recurrent vertigo, and others, have circulated for some 50 years, it was not until Neuhauser's landmark work in 2001 founding the first set of reliable criteria for what is now known as vestibular migraine that the disease began creeping into the physician's diagnostic repertoire [2, 3]. Even as late as 2010, skeptics voiced their denial of its existence [4]. Nevertheless, VM is now widely recognized as a distinct diagnostic entity by both the Barany Society and the International Headache Society (IHS), which jointly formulated the revised diagnostic criteria for VM in 2012 [5].

With an estimated lifetime prevalence of 1% and 1-year prevalence of 5% in women between the ages of 40 and 54, VM is likely the most common vestibular disorder [6–8]. It afflicts predominantly females, with a 5-fold increased risk, and has a mean age of onset of 38 in women and 42 in men [3]. VM accounts for about 10% of patients seen for migraine and about 10% of patients (although anecdotally up to 50% in the senior author's experience) seen for dizziness [9]. Episodes of vertigo, imbalance, dizziness, and/or disequilibrium seen in VM last from seconds to days and may or may not be temporally associated with migraine headaches, with or without aura [3, 5, 10]. The temporal association between VM and migraine headaches can also be inconsistent between patients and in the same patient [10, 11]. Often, vestibular symptoms in VM do not begin until several years after the onset of typical migraines, and, in some, there may be a headache-free interval of years before the onset of VM [10, 12, 13]. Despite VM's prevalence and new diagnostic criteria, its pathophysiology is unclear, its management is mostly anecdotal, and it remains a diagnosis of exclusion based only on history, without pathognomonic physical exam, laboratory, or imaging findings.

One of the reasons that physicians were so reluctant to accept VM as a distinct disease entity was that it produced so many symptoms overlapping with various well-established vestibular disorders such as Meniere disease (MD), benign paroxysmal positional vertigo (BPPV), and neurologic conditions that can elicit dizziness such as basilar migraine [5, 10, 13–17]. In some cases, VM may be indistinguishable from MD based on history and symptoms [1]. Furthermore, VM may be comorbid with MD, further confounding the diagnosis [15].

Meniere disease is characterized by vertigo associated with tinnitus, aural fullness, and/or hearing loss [18]. Its prevalence is 5 to 10 times lower than that of VM and has a lower female to male preponderance of 1.3 : 1 [12, 19]. Patients are affected usually in their 4th to 7th decades of life, often presenting with episodic vertigo with or without low-frequency hearing loss, though the disease course may be quite variable [18]. Like VM, the diagnosis of MD is based mainly on history, especially when audiological tests are normal; and its pathophysiology is also uncertain [19, 20].

One study found that of patients diagnosed with VM or MD in a tertiary dizziness clinic, about a quarter had symptoms which could have met criteria for either. Of the patients with VM alone, 38% presented with subjective hearing loss, aural pressure, and tinnitus during episodes of dizziness and headaches; and of the patients with MD alone, 49% presented with migrainous features such as photophobia and headache with vomiting or first degree relative to migraine [15]. MD patients have been found to be twice as likely to have migraine compared to those without MD [21]. And, interestingly, those with migraine are more likely to have earlier onset and bilateral hearing loss from MD [22, 23].

Clearly, the relationship between VM and MD is beyond that of chance alone. In this review, we explored the pathophysiology of these disorders and sought to clarify how the two disease processes are linked and why their presentations are so much alike.

## 2. Review

### 2.1. Current Theory of Migraine

Migraine is the common thread connecting VM and MD. Thus, the pathophysiology of migraine is crucial to the understanding of VM and MD. A brief discussion of the general concepts of migraine, especially aspects potentially important to the pathophysiology of VM and MD, is offered in lieu of a complete analysis of its mechanisms, as this study cannot do justice to the full scope of contemporary migraine theory.

The most widely accepted model of migraine resolves around the trigeminovascular system (TVS) [24, 25]. The TVS consists of the trigeminal nuclei, the trigeminal ganglion and nerve, and the meningeal vasculature which it innervates [24, 26]. More specifically, the ophthalmic division (V1) of the trigeminal nerve innervates dural and pial blood vessel nociceptors that, when activated, cause a release of vasoactive neuropeptides such as substance P, calcitonin gene-related peptide, and neurokinin A which in turn cause increased cerebral blood flow, release of proinflammatory factors, and a reaction called neurogenic inflammation [27]. The nociceptive afferent neurons project to what is called the trigeminocervical complex (TCC), consisting of the trigeminal nucleus caudalis in the brainstem and the spinal cord dorsal horns of C1 and C2. The TCC connects to the ventroposteromedial (VPM) and posterior nuclei of the thalamus, which relay information to the cerebral cortex, including the somatosensory cortex, the insular cortex, and the anterior cingulate cortex [24]. The TCC also has reciprocal connections with other parts of the brainstem, including the ventromedial medulla (VMM) and the ventrolateral periaqueductal gray (VLPAG), and hypothalamic areas. These areas are involved in nociceptive processing, and they also receive modulation from the cerebral cortex through descending pathways [24]. The activation of the TVS is how headaches are believed to occur in migraine.

What activates the TVS is uncertain. One hypothesis involves another popular but controversial theory, cortical spreading depression (CSD). CSD is a massive wave-like, slowly propagating depolarization of the brain that typically starts in the occipital cortex and leads to prolonged suppression of cortical activity [25]. CSD is triggered when the local concentration of certain ions reaches a threshold and activates NMDA receptors through release of glutamate from cortical pyramidal cells [28]. CSD is believed to be the cause of auras, which occur at a progression rate of a few minutes, similar to that of CSD, and include symptoms such as scotomas, hemianopsia, visual patterns, flashing lights, paresthesias, and hallucinations [24]. CSD disrupts transmembrane ionic gradients and increases extracellular concentrations of molecules that can activate meningeal nociceptors of the TVS as well as trigeminal nuclei of the TCC [29–31].

Another theory is that the TVS can be directly activated by the dysfunction and dysregulation of brainstem nociceptive nuclei, such as the VMM and VLPAG mentioned previously. Thereby, migraine headaches are triggered by direct activation of the TVS instead of through a sequential pathway starting from the meningeal nociceptors [24]. In summary, migraine can be seen as a pathological brain disease other than vascular or pain disorder, in which numerous pathways within the brain are activated and the central nervous system is oversensitized, making different systems, such as the vestibular system and the multisensory integrative routes it is involved in, prone to dysfunction [32].

### 2.2. How Migraine Produces Vestibular Symptoms

It has been postulated that the vestibular symptoms of VM are types of aura in migraines caused by the spread of CSD to the vestibular nuclei or cortices [13]. However, the vestibular symptoms in VM last from seconds to days, which do not fit the typical profile of auras. Also, it does not explain the unilateral vestibular canal paresis seen in some patients. When vertigo does occur like an aura, it is often associated with dysarthria, diplopia, ataxia, and altered consciousness, which is more diagnostic of basilar migraine [25].

Perhaps a better way to justify the relationship between VM and migraine is through vascular theories directly involving the TVS. It has been shown that the trigeminal nerve directly affects inner ear blow flow through the innervation of cochlear vasculature. Vass et al. demonstrated in Guinea pigs that the cochlea and the vestibular labyrinth receive trigeminal innervation from the ophthalmic branch, which provides parasympathetic innervation to the basilar and anterior inferior cerebellar arteries. Stimulation of the nerve led to increased vascular permeability and plasma protein extravasation [33–35]. Furthermore, Shore et al. showed that the trigeminal nerve also innervates the cochlear nucleus and the superior olivary complex [36]. A study in humans validated these results by showing that painful stimulation of the trigeminal nerve (through electrodes applied to the supraorbital point) elicited nystagmus or changes in preexisting nystagmus [37]. This provides a mechanism in which activation of the TVS, as in migraines, can cause peripheral vestibular dysfunction.

Central vestibular symptoms such as nystagmus during VM attacks and between attacks cannot simply be explained by ischemic events affecting the peripheral vestibular organs [16, 38]. A dysfunction of central processing of vestibular inputs along with other sensory information may be responsible for central vestibular findings in VM [24]. A specific vestibular cortex has yet to be discovered in humans [25]. However, vestibular information travels through and is processed in many areas including the ventroposterolateral (VPL) and VPM thalamus which are relay stations for visual and proprioceptive along with vestibular inputs. Activation of the thalamus shown through positron emission tomography (PET) and functional magnetic resonance imaging (fMRI) during migraine attacks suggests that the vestibular symptoms in VM are a result of faulty sensory integration [25, 29].

There are reciprocal connections between vestibular nuclei and nociceptive centers (e.g., VLPAG) and between vestibular nuclei and trigeminal nuclei (e.g., trigeminal nucleus caudalis), which can also explain vestibular symptoms in migraine patients [25, 39]. Other connections between vestibular nuclei and brainstem structures such as the parabrachial nucleus, raphe nuclei, and locus coeruleus may be responsible for sensitization of the TVS and nociceptive pathways during VM episodes [39–41]. The sensitization of thalamic pathways may be the cause of motion-sickness susceptibility in VM patients [42, 43]. Moreover, the degree of thalamic activation shown on fMRI during vestibular stimulation correlates with the frequency of migraine attacks in VM patients [25]. The reciprocity between the vestibular system and brain centers implicated in migraine is further demonstrated in another study, where rotational and caloric vestibular testing triggered migraines more in those with a history of migraine than in those without (49% versus 5%, resp.) [44]. Interesting, caloric stimulation has been reported to trigger migraine even in those without migraine history [45]. This shows that the peripheral vestibular system may have the ability to modulate migraines just as migraines may trigger vestibular dysfunction.

Anecdotally, the senior author has treated dizzy patients who presented with typical VM symptoms but have had histories typical of vestibular neuritis at the onset of their disease. These patients usually recount several days of severe, debilitating vertigo which resolves gradually but is never fully compensated and sometimes evolves into chronic dizziness/imbalance, along with migraine features like photophobia and phonophobia. Furthermore, in a yet unpublished study, we found that while examining the effects of intratympanic gentamycin injection on MD patient with or without migraine, migraines and vestibular migraines were triggered after chemical ablation of the vestibular system with gentamycin injections in several patients with migraine history but not in those without. Thus, our experience appears to be consistent with the previous studies, suggesting that insult to the peripheral vestibular system may be projected centrally.

Further evidence that VM is a disease of multisensory integration in the central nervous system was seen in a study of tilt perception in VM, migraine, and normal patients. When the 3 groups of patients sat in a centrifuge and an interaural centrifugal force was generated, the development of the perception of tilt in the roll plane was slower in the VM group compared to the other 2, but eye movement responses such as rotational axis shift were the same in all groups [46]. In other roll-type experiments, VM patients were found to have decreased motion perception thresholds when canal and otolith signals were presented simultaneously, but perception of tilt was slower when the signals were conflicting [47]. This may be a potential origin for motion sensitivity in VM patients. The authors believe that these results suggest VM is, at least in part, a result of dysfunctional vestibular signal integration and abnormal sensitization possibly of cerebellar origin [46, 47].

### 2.3. Pathophysiology of Meniere Disease

Similar to VM, although with a more extensive history of recognition and research, the pathophysiology of MD has yet to be fully elaborated [48]. The most well-known aspect of MD is the presence of endolymphatic hydrops (ELH), a hallmark of the disease which can be confirmed by pathology postmortem [20, 49]. However, even though ELH was found in all patients with MD in a study of temporal bones (28 patients), only 51 of 79 patients (65%) found to have ELH had MD [50]. Furthermore, ELH can be found in autoimmune inner ear disease, posttraumatic ears, otosyphilis, otosclerosis, endolymphatic sac tumors, and other disorders [20]. This brings about the question whether ELH is the cause of MD or if it is a byproduct of another process which causes both MD and ELH. It would seem that ELH is necessary but not sufficient for the development of MD.

Endolymphatic hydrops is a consequence of a disturbance in the homeostasis of endolymph. Some believe that MD is a result of a “fragile” ear in which certain factors make the inner ear more prone to dysregulation of homeostasis and more susceptible to changes in the body or the environment [48]. These changes may include stress, sleep deprivation, diet change, hormonal alterations, allergies, barometric pressure changes, or any of the diseases listed previously to be associated with ELH [20, 48]. The factors that actually make the ear vulnerable, however, are unclear.

Older theories of ELH postulated that obstructions to the flow of endolymph, from its production by the stria vascularis, passing through the endolymphatic duct (ELD), to its resorption in the endolymphatic sac (ELS), lead to the development of ELH [49]. This is supported by the observation of ELH in patients with ELD obstruction or ELD hypoplasia [20]. However, these conditions are not necessary for the presence of ELH, and the ionic homeostasis of endolymph does not seem to be significantly affected by this flow pathway [51]. Therefore, the theory of radial flow, wherein endolymph is both produced and absorbed in the cochlear duct, arose [20]. In this theory, molecules and channels such as aquaporins and gated ionic channels regulate the composition and volume of endolymph [52]. This may be why diuretics have shown some evidence of effectiveness in treating MD, though the effect has not been proven to be great [53].

Damage to the spiral ganglion by chronic excitotoxicity has been theorized to be the cause of gradual hearing loss in MD [20]. In this model, ELH in the cochlea results in the release of excitatory mediators such as glutamate and other amino acids, which can eventually lead to neuronal toxicity and death [54]. This process involves the apoptosis cascade, in which reactive oxygen species (ROS) are produced and a caspase-dependent pathway is activated, followed by cell death [54]. More specifically, in hydropic conditions, elevated levels of a glutamate aspartate transporter (GLAST) in the cochlea increase glutamate presence, which activates N-methyl-D-aspartate (NMDA) receptors and subsequently activate nitric oxide synthase (NOS) [55, 56]. Nitric oxide produced by NOS combines with superoxide to create peroxynitrate, which is a potent ROS capable of cytotoxic neuronal apoptosis [54, 57]. In Guinea pig models, surgically induced hydrops reduced 8th cranial nerve diameter and caused differential nerve damage in that neuronal loss was greater than inner hair loss at the apex, consistent with typical low-frequency hearing loss in MD. However, the degree of hydrops did not correlate with the degree of reduction in 8th nerve diameter, and when aminoguanidine (an inhibitor of NOS) was used, there was no evidence of neuronal protection [58, 59]. Furthermore, the true relationship between ELH and neuronal damage is yet to be proven and can only be extrapolated to humans.

### 2.4. Comparing and Contrasting Vestibular Migraine and Meniere Disease

Prosper Meniere suggested a link between migraine and MD as early as 1861, but a definitive association has yet to be established [21]. Radtke et al. found the lifetime prevalence of migraine to be 56% in MD patients, compared to 25% in controls [21]. However, Gopen et al. found the incidence of migraine in MD to be 4.5%, which was not significantly different from those without MD (3.8%) [60]. Studies have supported both sides of the argument [61–64]. Nevertheless, there is an unequivocal overlap between many symptoms of VM and MD. Ghavami et al. found that, in 37 patients with definite MD, 95% had one or more features of migraine, 51% had migraine headaches, and 48% met the IHS criteria for diagnosis of VM [61].

MD patients typically have fluctuating aural symptoms of hearing loss, tinnitus, and aural fullness [18]. However, aural pressure may resemble headache and patients with migraine may also have tinnitus and hearing loss [65]. Phonophobia is a prominent symptom in VM but can commonly occur in MD [65]. The vestibular symptoms of VM are broadly defined to be vertigo, dizziness, or other imbalances lasting from 5 minutes to 72 hours, which can easily describe the type of vertigo seen in MD patients [5]. In fact, vertigo or dizziness occurs in migraine patients 25–35% of the time, with many episodes closely resembling MD attacks [2].

Based on the diagnostic criteria of both VM and MD, hearing loss stands out as a potential differentiating factor between the two disorders [5, 18]. Patients with definite MD must have audiograms showing low-frequency to medium-frequency hearing loss [18]. However, hearing loss is not a requirement in probable MD, and many normal-hearing patients may develop hearing loss over time to be later diagnosed with definite MD [66]. Moreover, hearing loss can occur in VM. The mechanism can be explained by the previously mentioned animal studies demonstrating the trigeminovascular control of inner ear blood flow, which could involve cochlear perfusion as well as vestibular end-organ perfusion [34, 67]. Accordingly, reports have shown that fluctuating hearing loss or progressive sensorineural hearing loss can occur in up to 25% of migraine patients [68, 69].

As seen in the previous discussion on the pathophysiology of VM and MD, MD is a disorder of the peripheral vestibular end-organs, while VM is one of both central and vestibular dysfunctions originating from a central occurrence. How can the two be related? One study found that cervical and ocular vestibular-evoked myogenic potential (VEMP) responses from VM and MD patients were found to resemble each other and that no one test could differentiate between the two disorders [70]. Furthermore, caloric testing and motion-sensitivity questionnaires have been shown to be unsuccessful in distinguishing between VM and MD [43]. It has been suggested that the inability to discriminate between the two diseases by clinical testing is evidence of a common origin [61]. Some have hypothesized that ELH may result from end-organ damage to the inner ear caused by VM and that VM and MD in fact share the same etiology [70]. Other authors have found that family history is frequently positive for both VM and MD; and they believe that a heritable syndrome with variable expression of both VM and MD features exists [22]. It appears that the triggers suggested for ELH as mentioned previously (stress, sleep deprivation, diet change, etc.) are similar to those for migraine [48]. This could be another mechanism in which VM and MD are linked.

One common origin for VM and MD has been thought to be related to chronic underperfusion leading to end-organ damage [61]. Decreased perfusion of the spiral modiolar artery, which primarily supplies the middle and apical turns of the cochlea, has been thought to cause low-frequency hearing loss, as in that of typical MD [71]. One hypothesis is that chronic recurrent damage from vasospasm of the spiral modiolar artery causes the ELH found in MD [61]. By this proposed pathway, the TVS in migraine patients causes perfusion deficits in the inner ear, and based on individual anastomotic patterns, different parts of the inner ear are affected [61]. For example, involvement of the spiral modiolar artery would lead to the historically described “cochlear” MD with low-frequency hearing loss; additional involvement of the vestibular arteries would lead to typical MD findings with vertigo and hearing loss; and involvement of the cochlear artery (found to primarily perfuse the basal cochlea) would result in atypical high-frequency hearing loss in MD [61, 71]. The different types of hearing loss have been seen in representative audiograms that can be peaked or flat [68, 72]. Thereby, the different hearing loss and vertigo variations seen in MD represent phenotypes derived from differential injury to the inner ear based on development of its vasculature. Although this suggested mechanism for migraine being the predecessor of both VM and MD is unproven, based on their experience, the authors recommend treating patients who have both MD and migraine but do not fulfill the criteria for VM with migraine prophylactic agents before intratympanic or more invasive surgical therapy [61]. In fact, our unpublished data showed that, after intratympanic gentamycin injection, the quality of life in MD patients with migraine is much poorer than that in those without migraine, although the controls of major vertigo attack are similar in both groups of patients.

One way to discover whether VM and MD share a common etiology is by examining the evolution in symptomatology and findings over time to see where they deviate or converge. In VM, hearing loss may be present but is typically bilateral [73]. In MD, however, bilateral hearing loss is rare at its onset [74]. Cochlear symptoms of tinnitus, aural fullness, and hearing loss have been found to become more prevalent (from 15% to 49%) in a study of VM patients over an average of 9 years [75]. However, hearing levels in MD patients decrease to a mean of 50–60 decibels in 5–10 years, while hearing loss remains mild in VM patients and decreases much slower than that of MD patients [75]. The majority of VM patients still suffer from vertigo in the long run, while MD patients seem to experience fewer vertigo episodes. In one study, 87% of VM patients had recurrent vertigo after an average of 9 years with frequency reduced in 56%, increased in 29%, and unchanged in 16% [75]. In a study of 119 MD patients after at least 14 years, vertigo completely resolved in 50% and somewhat resolved in 28% [76]. In a review of retrospective studies totaling nearly 8000 MD patients, the overall frequency of vertigo attacks seemed to decrease [74]. There were some reports of patients who did not fare as well, however, and suffered from increasing severity or frequency of vertigo. In general, vertigo attacks in MD patients seemed to vary widely in terms of frequency and severity [74]. In VM, deterioration of vestibular function over time has been reported, evidenced by increased vestibular abnormalities such as central oculomotor dysfunction between episodes in about half of patients over nearly a decade [75, 77]. In MD, caloric tests have shown a general increase in hyporesponsiveness over time [74]. But, again, there was great variation in caloric results in patients with disease progression [74].

The reason VM and MD look so much alike in some cases, yet quite dissimilar in others, is that we do not yet truly understand the pathophysiology of either disease. And thus we use diagnostic criteria which are purposefully modest in specificity to encompass a broad spectrum of presentations. The arguments for a common etiology for VM and MD are provocative and feed our desire for an all-encompassing solution, but there is evidence that aspects of VM and MD can be very different from one another. As of yet, it is unclear if migraine or another trigger is the initiator of both VM and MD, if VM causes MD, if MD causes VM, or if the two simply occur in parallel due to an indirect cause with many degrees of separation. It could very well be, as we learn more about the vestibular disorders, that different subtypes of VM and MD will be parsed out.

While performing research on either disorder, we must keep in mind that previous work was performed when VM was not fully recognized and that it was (and is) often difficult to distinguish between VM and MD such that patients may have been mislabeled and conclusions were drawn based on historical diagnostic criteria. It may be that we will not be able to make significant strides in understanding either disease until new technology allows for novel pathways of experimentation, but we have taken important steps in recent years such as the use of new MRI protocols to image migraine and ELH in MD [78–80]. We do not wish to dishearten physicians who treat patients with dizziness by suggesting that VM and MD are far too difficult currently to distinguish and properly diagnose. In fact, we believe that the diagnostic criteria for* definite* VM and* definite* MD are clear and useful and that patients often can be appropriately classified into either category [5, 18]. The difficulty lies within the* probable* categories, which leave much to be desired in achieving specificity in differentiating between VM and MD. Redefined criteria, which will take more research to develop, are clinically imperative for the future. Our need to assign diagnoses even to patients with uncertain ailments and our need to categorize illnesses for the sake of satisfying patients' desires for therapy have put us in a position of both authority and skepticism.

## 3. Conclusion

The pathophysiology of vestibular migraine and Meniere disease has yet to be completely defined. As such, there is a great deal of overlap between the two disorders in terms of presentation and diagnostic criteria. Migraine is believed to be the cause of vestibular migraine through effects on the inner ear from the trigeminovascular system and through direct central activation of vestibular centers. While endolymphatic hydrops is still thought to be the cause of Meniere disease, this theory has been challenged and migraine has been implicated as a common etiology between vestibular migraine and Meniere disease. There is not enough evidence to definitively link vestibular migraine and Meniere disease in a mechanistic way, but awareness of the blurred lines between the two disorders may assist in clinical diagnosis and treatment.
